# *Clethra fimbriata* hexanic extract triggers alteration in the energy metabolism in epimastigotes of *Trypanosoma cruzi*

**DOI:** 10.3389/fmolb.2023.1206074

**Published:** 2023-09-25

**Authors:** Daniel Pardo-Rodriguez, Paola Lasso, Mary Santamaría-Torres, Mónica P. Cala, Concepción J. Puerta, Jonh Jairo Méndez Arteaga, Jorge Robles, Claudia Cuervo

**Affiliations:** ^1^ Grupo de Enfermedades Infecciosas, Pontificia Universidad Javeriana, Bogotá, Colombia; ^2^ Grupo de Fitoquímica, Pontificia Universidad Javeriana, Bogotá, Colombia; ^3^ Grupo de Productos Naturales, Universidad del Tolima, Tolima, Colombia; ^4^ Metabolomics Core Facility—MetCore, Vice-Presidency for Research, Universidad de los Andes, Bogotá, Colombia; ^5^ Grupo de Inmunobiología y Biología Celular, Pontificia Universidad Javeriana, Bogotá, Colombia

**Keywords:** Chagas disease, hexanic extract of Clethra fimbriata, energy metabolism, multiplatform untargeted metabolomics, triterpenes, Trypanosoma cruzi

## Abstract

Chagas disease (ChD), caused by *Trypanosoma cruzi*, is endemic in American countries and an estimated 8 million people worldwide are chronically infected. Currently, only two drugs are available for therapeutic use against *T. cruzi* and their use is controversial due to several disadvantages associated with side effects and low compliance with treatment. Therefore, there is a need to search for new tripanocidal agents. Natural products have been considered a potential innovative source of effective and selective agents for drug development to treat *T. cruzi* infection. Recently, our research group showed that hexanic extract from *Clethra fimbriata* (CFHEX) exhibits anti-parasitic activity against all stages of *T. cruzi* parasite, being apoptosis the main cell death mechanism in both epimastigotes and trypomastigotes stages. With the aim of deepening the understanding of the mechanisms of death induced by CFHEX, the metabolic alterations elicited after treatment using a multiplatform metabolomics analysis (RP/HILIC-LC-QTOF-MS and GC-QTOF-MS) were performed. A total of 154 altered compounds were found significant in the treated parasites corresponding to amino acids (Arginine, threonine, cysteine, methionine, glycine, valine, proline, isoleucine, alanine, leucine, glutamic acid, and serine), fatty acids (stearic acid), glycerophospholipids (phosphatidylcholine, phosphatidylethanolamine and phosphatidylserine), sulfur compounds (trypanothione) and carboxylic acids (pyruvate and phosphoenolpyruvate). The most affected metabolic pathways were mainly related to energy metabolism, which was found to be decrease during the evaluated treatment time. Further, exogenous compounds of the triterpene type (betulinic, ursolic and pomolic acid) previously described in *C. fimbriata* were found inside the treated parasites. Our findings suggest that triterpene-type compounds may contribute to the activity of CFHEX by altering essential processes in the parasite.

## 1 Introduction

The parasite *Trypanosoma cruzi* is the etiological agent of Chagas disease (ChD), a neglected tropical disease that affects more than 8 million people worldwide (WHO, 2021). Although, ChD is endemic to American countries, population movement has led to its spread to non-endemic countries (Klein et al., 2012), making it a global public health concern (Rassi et al., 2010; Tarleton, 2016; Pérez-Molina and Molina, 2017). In Colombia, it has been reported that approximately 436,000 people are infected and that about 11% of the population is at risk of acquiring the infection (Rassi et al., 2010; World Health Organization, 2015; Olivera et al., 2019).

There is no vaccine available for ChD, but there have been two drugs used since the 1970s: Nifurtimox (NFX) and Benznidazole (BNZ) (Nunes et al., 2013; Bustamante and Tarleton, 2014; Bern, 2015). However, the difficulty in conducting clinical trials during the chronic phase of the infection has made it challenging to determine the effectiveness of treatment during this phase (Urbina, 2010; Urbina, 2015). Additionally, the treatment with NFX and BNZ is associated with several issues, including high toxicity, side effects and a prolonged treatment time (Bern, 2015; Manne-Goehler et al., 2016). Furthermore, the presence of *T. cruzi* isolates with different degrees of susceptibility to these drugs has been reported (Castro et al., 2006; Mejía-Jaramillo et al., 2012). In addition to this, it has been found that *T. cruzi* has the ability to enter a dormant state known as non-replicating amastigote, which allows the parasite to resist the pharmacological stress induced by BNZ (Sánchez-Valdéz et al., 2018). Therefore, the development of safer and more efficient therapeutic alternatives for the treatment of ChD is essential.

Plants have been used for a long time in the treatment of multiple diseases and recently have gained renewed interest as a starting point to propose new natural products with specific bioactivities (Schmidt et al., 2012; Lopera Valle et al., 2013). In fact, an estimated 60% of currently available drugs are derived from natural products (Newman and Cragg, 2016; Newman and Cragg, 2020), suggesting the importance of natural sources in drug discovery. Colombia’s distinct geographical location affords a varied range of ecosystems that support one of the world’s highest diversity and dispersion of animals, fungi, and plants (Gori et al., 2022). As a result, the country is considered a promising source of chemical structures with specific biological activities.

Our research group recently evaluated the trypanosomicidal effects of the native Colombian plant *Clethra fimbriata*, finding that the ethanolic and hexanic extracts are effective against the different stages of *T. cruzi*. Further, the extracts induce the production of cytokines and cytotoxic molecules in CD4^+^ and CD8^+^ T cells from healthy donors, an effect that may be associated with the high content of pentacyclic triterpenes found in *C. fimbriata* (Castañeda et al., 2021; Pardo-Rodriguez et al., 2022). Based on these findings, this research focused on associating metabolic alterations and death mechanisms induced after treatment of epimastigotes with the CFHEX extract, using a multiplatform untargeted metabolomics approach.

## 2 Materials and methods

### 2.1 Plant material and extraction

Plant material was collected under the “Permit for wild species specimen collection of biological diversity for research with non-commercial purposes” (Permiso marco de recolección de especímenes de especies silvestres de la diversidad biológica para investigación con fines no comerciales) granted to the Pontificia Universidad Javeriana (Resolution 778 of 7 July 2017) issued by the “National Environmental Licensing Authority”. *C. fimbriata* was collected in Majuy Hill, Via Cota, Cundinamarca, Colombia, and taxonomically identified by the Colombian National Herbarium (voucher specimen number COL 610805). *C. fimbriata* aerial parts were dried and crushed, followed by extraction by successive maceration (Five extractions) with 1:10 sample to solvent ratio, using hexane (CFHEX). Obtained extract was concentrated by evaporation *in vacuo*. Prior to biological tests, the extract was resuspended in ethanol.

### 2.2 Parasite maintenance

*T. cruzi* Y-strain epimastigotes (MHOM/BR/00/Y); a discrete typing unit (DTU TcII) (Pavia et al., 2012), were maintained in the exponential growth phase in Liver Infusion Tryptose (LIT) medium supplemented with 15% heat inactivated fetal bovine serum (FBSi) (Eurobio), 100 U/mL penicillin and 100 μg/mL streptomycin (Eurobio), at 26°C.

### 2.3 Sample preparation

Epimastigotes of *T. cruzi* were cultured in LIT medium supplemented with 15% FBSi at 26 °C. Once they reached an exponential growth phase, 1 × 10^8^ parasites were transferred to fresh culture medium, and incubated with the IC_90_ (690 μg/mL) of the CFHEX for 36 h. As a negative control the parasites were incubated with fresh culture medium. After the incubation time, the parasites were washed three times with phosphate buffered saline (PBS) at 4 °C and immediately frozen in liquid nitrogen and kept at −80 °C until further processing. Each treatment was evaluated in six independent biological replicates.

#### 2.3.1 Metabolite extraction

For the extraction of metabolites from the treated and control parasites, 500 µL of a solution of MeOH-water (4:1 v/v) were added to each of the samples. Then, two-3 mm tungsten carbide beads were added to each of the cryovials and vortexed at 3,200 rpm for 1 min. Subsequently, the samples were taken to the Tissue Lyser to perform 5 cycles of 30 Hz for 1 min. The samples were centrifuged at 15,700 *g*, at 4 °C for 20 min and filtered through 0.22 µm filters. Finally, 200 µL of the extracts were taken, which were used for subsequent analysis by reverse phase liquid chromatography coupled to mass spectrometry with a time-of-flight analyzer (RP-LC-QTOF/MS) in positive and negative polarity and hydrophilic interaction chromatography coupled to mass spectrometry with a time-of-flight analyzer (HILIC-LC-QTOF-MS) (Rojo et al., 2015).

#### 2.3.2 Untargeted metabolomics by RP-LC-QTOF-MS

Samples were analyzed using an Agilent Technologies 1,260 liquid chromatography system coupled to a 6545 Q-TOF quadrupole time-of-flight mass analyzer with electrospray ionization. Five µL of the sample were injected onto a C_18_ column (InfinityLab Poroshell 120 EC-C18 (100 × 3.0 mm, 2.7 µm)) at 30 °C and compound gradient elution: 0.1% (v/v) of formic acid in Milli-Q^®^ water (Phase A) and 0.1% (v/v) of formic acid in acetonitrile (Phase B) with a constant flow of 0.4 mL/min. The elution gradient started at 25% with respect to B and increased over 35 min to 95% B. Finally, the gradient decreased to 36% B over a period of 1 min and was maintained for a further 9 min until the system was rebalanced. Detection by mass spectrometry was performed in positive and negative ESI mode in full scan from 100 to 1,100 m*/z*. Throughout the analysis, two reference masses were used for mass correction: *m/z* 121.0509 [C_5_H_4_N_4_]^+^, *m/z* 922.0098 [C_18_H_18_O_6_N_3_P_3_F_24_]^+^ in positive mode and *m/z* 112.9856 [C_2_O_2_F_3_(NH_4_)]^-^, *m/z* 1,033.9881 [(C_18_H_18_O_6_N_3_P_3_F_24_+ trifluoroacetic acid)-H]^-^ in negative mode.

#### 2.3.3 Untargeted metabolomics by HILIC-LC-QTOF-MS

Two µL of the sample was injected onto a Kinetex HILIC 100 A column (150 × 3.0 mm, 2.6 µm) at 40 °C and a gradient elution composed of: 10 mM ammonium acetate in acetonitrile:water (50:50) (Phase A) and 10 mM ammonium acetate in acetonitrile: water (95:5) (Phase B) with a constant flow of 0.4 mL/min. The elution gradient started at 99% with respect to B and decreased for 15 min until reaching 50% B, where it was maintained for 1 min. Finally, the gradient increased to 99% B and was maintained for an additional 6 min until the system re-equilibrated. Detection by mass spectrometry was performed in negative ESI mode in full scan from 50 to 1,100 m*/z*. Throughout the analysis, two reference masses were used for mass correction: *m/z* 112.9856 [C_2_O_2_F_3_(NH_4_)]^-^ and *m/z* 1033.9881 [C_18_H_18_O_6_N_3_P_3_F_24_+trifluoroacetic acid)-H]^-^.

#### 2.3.4 Untargeted metabolomics by GC-QTOF-MS

One hundred µL of the extracts were dried in a speedvac for 3 h at 35 °C. 10 μL of O-methoxyamine in pyridine (15 mg/mL) were added and vortexed at 3,200 rpm for 10 min. Subsequently, samples were kept in the dark for 16 h and 10 µL of *N,O*-Bistrifluoroacetamide with 1% trimethylsilyl chloride were added and incubated at 70 °C for 1 h. Finally, the samples were allowed to cool to room temperature for 30 min, 100 µL of methyl stearate in heptane as internal standard (10 mg/L) were added and the blend was vortexed for 10 min at 3,200 rpm. The derivatized samples were immediately analyzed according to the following methodology.

For data acquisition, an Agilent Technologies 7890B gas chromatograph coupled to an Agilent Technologies GC/Q-TOF 7250 time-of-flight mass selective detector, equipped with a split/splitless injection port (250 °C, ratio split 30) and an Agilent Technologies 7693A autosampler. The electron ionization (EI) source was operated at 70 eV. An Agilent Technologies J&W HP-5MS column (30 m, 0.25 mm, 0.25 µm) was used. The carrier gas flow was helium at a constant flow of 0.7 mL/min. The oven temperature was programmed from 60 °C (1 min) to 325 °C (10 min). The temperature of the transfer line to the detector, the source filament and the quadrupole were maintained at 280 °C, 230 °C and 150 °C, respectively. Detection by mass spectrometry was carried out between 50 and 600 m*/z* at a speed of 5 spectra/min.

#### 2.3.5 Quality controls samples (QC)

Quality control (QC) samples were prepared by mixing equal volumes of the metabolic extract from each sample. Subsequently, the preparation and analysis of the QC samples were performed following the procedures described above in each of the analytical platforms. To determine the reproducibility of sample preparation and the stability of the analytical platform used, several QC elutions were performed until the analytical system equilibrated. Subsequently, the QC samples were analyzed every three randomly injected samples.

### 2.4 Data processing and analysis

The data obtained by LC-MS was processed using the Agilent MassHunter Profinder program for deconvolution, alignment and integration of the data using the recursive and molecular feature extraction algorithms. Treatment of the GC-MS data obtained consisted of deconvolution and identification of the metabolites using the Agilent MassHunter Unknowns Analysis program and the Fiehn and NIST libraries. Then the alignment of the retention times was carried out in the Agilent Mass Profiler Professional program, the results were exported to the Agilent MassHunter Quantitative program for data integration. Finally, the data obtained from GC-MS and LC-MS data processing were manually inspected. Then, the data was filtered by presence and by reproducibility, keeping only the metabolites present or absent in 100% of the samples belonging to the same group and with a coefficient of variation in the QC of less than 20%.

The identification of the molecular characteristics with statistically significant differences between the two groups (treated and untreated parasites) was carried out using univariate (UVA) and multivariate (MVA) statistical analysis. Regarding the UVA analysis, the *p-value* was determined by nonparametric tests (Mann-Whitney U test) using SIMCA-P + 16.0 (Umetrics). For the MVA analyses, an unsupervised principal component analysis (PCA) was performed to observe the unsupervised distribution of the analyzed samples. Subsequently, supervised orthogonal partial least squares discriminant analysis (OPLS-DA) models were performed to select the molecular features responsible for the separation between the groups. The MVA was performed using the SIMCA-P+16.0 software (Umetrics). The selected statistically significant molecular characteristics met at least one of the following requirements: 1) UVA: *p-value* < *0.05* and 2) MVA: Variance Important in Projection (VIP) > 1.

#### 2.4.1 Annotation of statistically significant molecular features

The metabolites obtained by GC-MS analysis were identified using the Fiehn version 2013 libraries and MassHunter Personal Compound Database and Library Manager Software B.08.00. Whereas the significant characteristics obtained by LC-MS were putatively assigned in the CEU Mass Mediator annotator (http://ceumass.eps.uspceu.es) by matching the exact observed mass of each compound with the available *m/z* values. online at METLIN (http://metlin.scripps.edu), KEGG (http://genome.jp/kegg), and LIPIDMAPS (http://lipidMAPS.org), using the following likely adducts: [M + H]^+^, [M + H-[H_2_O]]^+^, [M + Na]^+^, and [M-H]^−^, [M + Formic acid-H]^−^, [M + Cl]^−^, [M-H-[H_2_O]]^−^ for positive and negative ionization modes, respectively. Furthermore, to confirm the identity of the metabolite, MS/MS analysis was performed.

#### 2.4.2 Altered metabolite pathway mapping

The analysis of the affected metabolic pathways in the treated parasites was performed using the “Pathway Analysis” tool of the MetaboAnalyst 5.0 server (http://www.metaboanalyst.ca/). For which, the altered compounds were annotated and compared with the *Trypanosoma brucei* (KEGG) metabolome available on the same server.

## 3 Results

**FIGURE 1:**
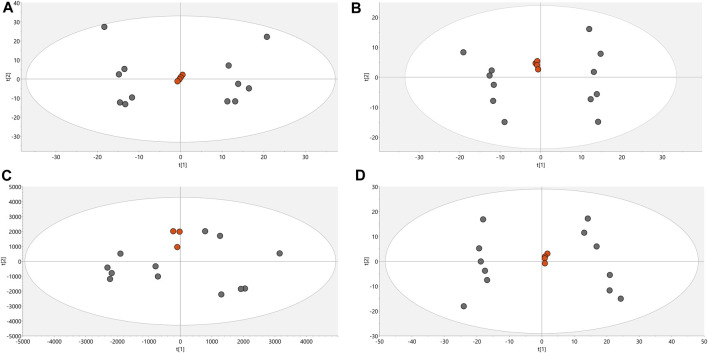
PCA score charts. **(A)**. LC/MS (+) *R* ^2^: 0.662, Q^2^: 0.323. **(B)**. LC/MS (−) *R* ^2^: 0.841, Q^2^: 0.654. **(C)**. GC/MS(+) *R* ^2^: 0.532, Q^2^: 0.177. **(D)**. HILIC/MS(−) *R* ^2^: 0.813, Q^2^: 0.66. Dots in orange color denote quality control, gray dots correspond to samples.

**FIGURE 2:**
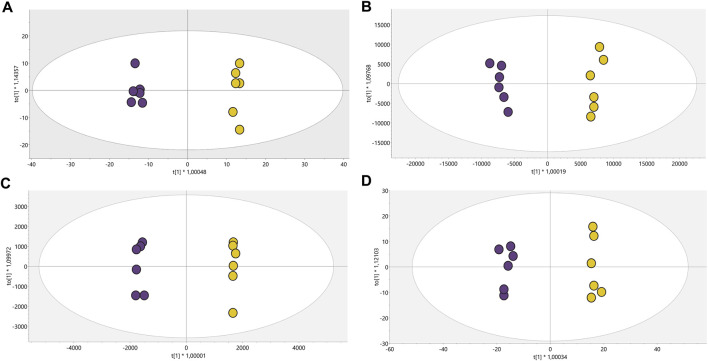
OPLS-DA models with Pareto scaling for metabolic analysis of treated parasites and control group. **(A)**. LC/MS (+): *R* ^2^: 0.402, Q^2^: 0.961, CV ANOVA: 5.2e^−5^, **(B)**. LC/MS(−): *R* ^2^: 0.789, Q^2^: 0.976, CV ANOVA: 1.0e^−5^, **(C)**. GC/MS: *R* ^2^: 0.715, Q^2^: 0.978, CV ANOVA: 5.7e^−4^, **(D)**. HILIC/MS: *R* ^2^: 0.713, Q^2^: 0.982, CV ANOVA: 3.2e^−6^. Dots in yellow color denote parasites treated with CFHEX and the violet dots the control group (untreated parasites).

Conditions associated with exposure to CFHEX (IC_90_: 690 μg/mL) in the epimastigote stage of *T. cruzi* were evaluated using untargeted metabolomics analysis. A multiplatform approach was used to detect the largest possible number of altered metabolites. The performance of the different analytical platforms was evaluated using unsupervised PCA models. The inspection of the clusters evidenced the clear grouping of the samples belonging to the quality control in the different analytical platforms used (Figure 1, orange dots). After verifying the performance of each analytical platform, the supervised orthogonal partial least squares regression method (OPLS-DA) was implemented to maximize the differences between the group made up of treated parasites and the group of untreated parasites (Figure 2) and to identify the molecular features with greater weight in the separation of the groups. The Pareto scaling method was used before the statistical analysis.

The OPLS-DA scoring plot presented in Figure 2 showed a clear separation between the groups: CFHEX-treated parasites (yellow dots) and untreated parasites (violet dots). Likewise, the variables *R* ^2^ and Q^2^, which measure the goodness of fit and the predictive capacity of the model developed from the data matrix, respectively, show adequate settings (*R* ^2^ > 0.402) and good predictive capacity (Q^2^ > 0.961) (Triba et al., 2015). Finally, to evaluate the reliability of the models, the variance cross-validation (CV-ANOVA) was performed, evidencing highly significant models in the four platforms analyzed (CV-ANOVA <0.05) (Eriksson et al., 2008). The individual differentiating metabolites were determined by a combination of MVA (VIP>1) and UVA (*p < 0.05*), obtaining a total of 154 altered compounds in the treated parasites, of which 25.16%, 30.97%, 14.84% and 12.26% were identified by LC/MS−, LC/MS+, GC/MS and HILIC/MS, respectively. In addition, the identification of some metabolites was achieved simultaneously by several platforms: 11.61% of compounds were found by LC/MS^−/+^, 1.29% by LC/MS^+^ and GC/MS, 1.94% by LC/MS^−^ and HILIC/MS, and 1.94% by LC/MS^−/+^ and HILIC/MS.

**FIGURE 3:**
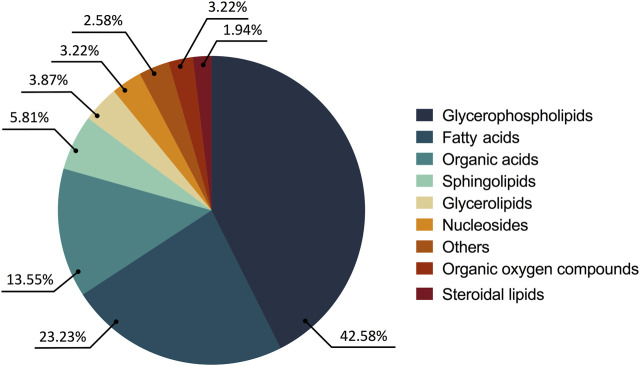
Altered metabolites in the treated parasites according to their chemical classes. The chemical classes are shown according to the color code.

The consolidated analysis of the metabolic modifications of the treated parasites showed 57.41% of the metabolites increased and 42.58% decreased. From these, 77.41% of the fluctuations were related to lipid chemical classes as follows: glycerophospholipids (65 compounds, 42.58%), fatty acids (36 compounds, 23.22%), sphingolipids (9 compounds, 5.81%), glycerolipids (6 compounds, 3.87%) and steroidal lipids (3 compounds, 1.93%). The remaining 22.58% of the changed metabolites belonged to chemical classes such as organic acids (21 compounds, 13.54%), nucleosides (5 compounds, 3.22%), oxygenated organic compounds (4 compounds, 2.58%), among others (5 compounds, 3.22%) (Figure 3).

**FIGURE 4:**
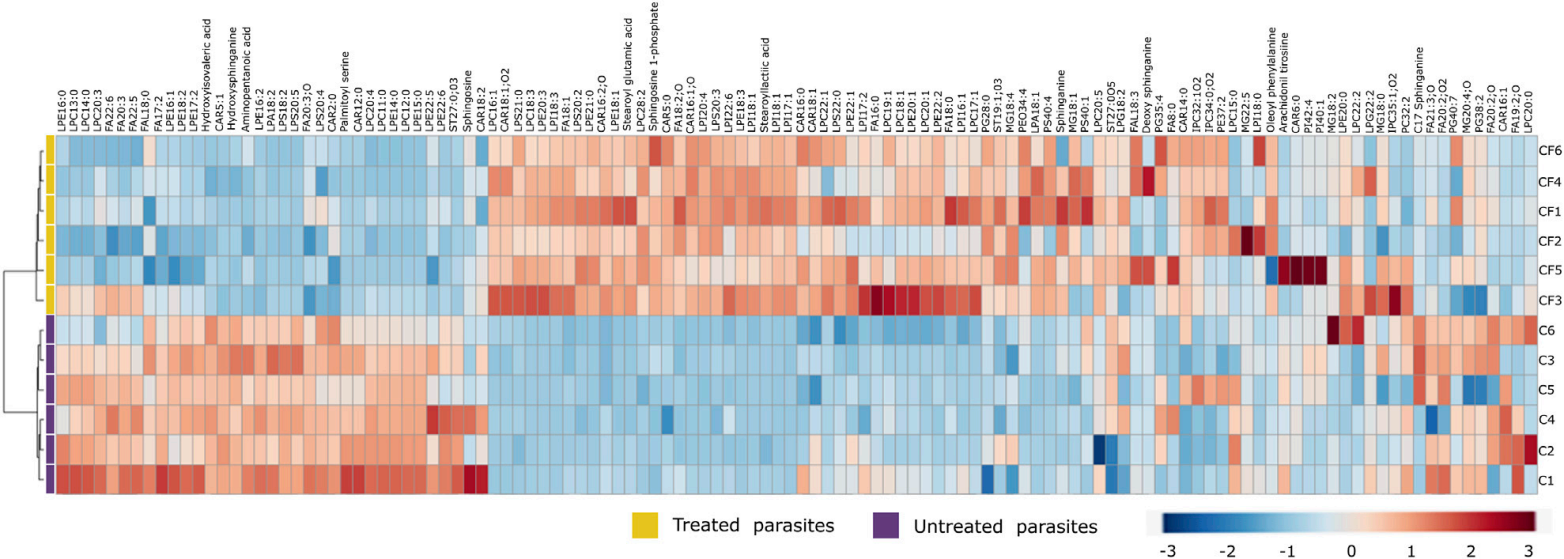
Heat map of lipid metabolites with statistically significant variation between *Trypanosoma cruzi* epimastigotes treated with CFHEX and the control group. The columns correspond to each altered metabolite identified, the rows correspond to the analyzed samples divided into the clades: CFHEX-treated parasites (yellow) and untreated parasites (violet). The level of variation is indicated on the right side on a color intensity scale representing relative abundance, where red colors denote metabolite increase and blue colors denote metabolite decrease.

**FIGURE 5:**
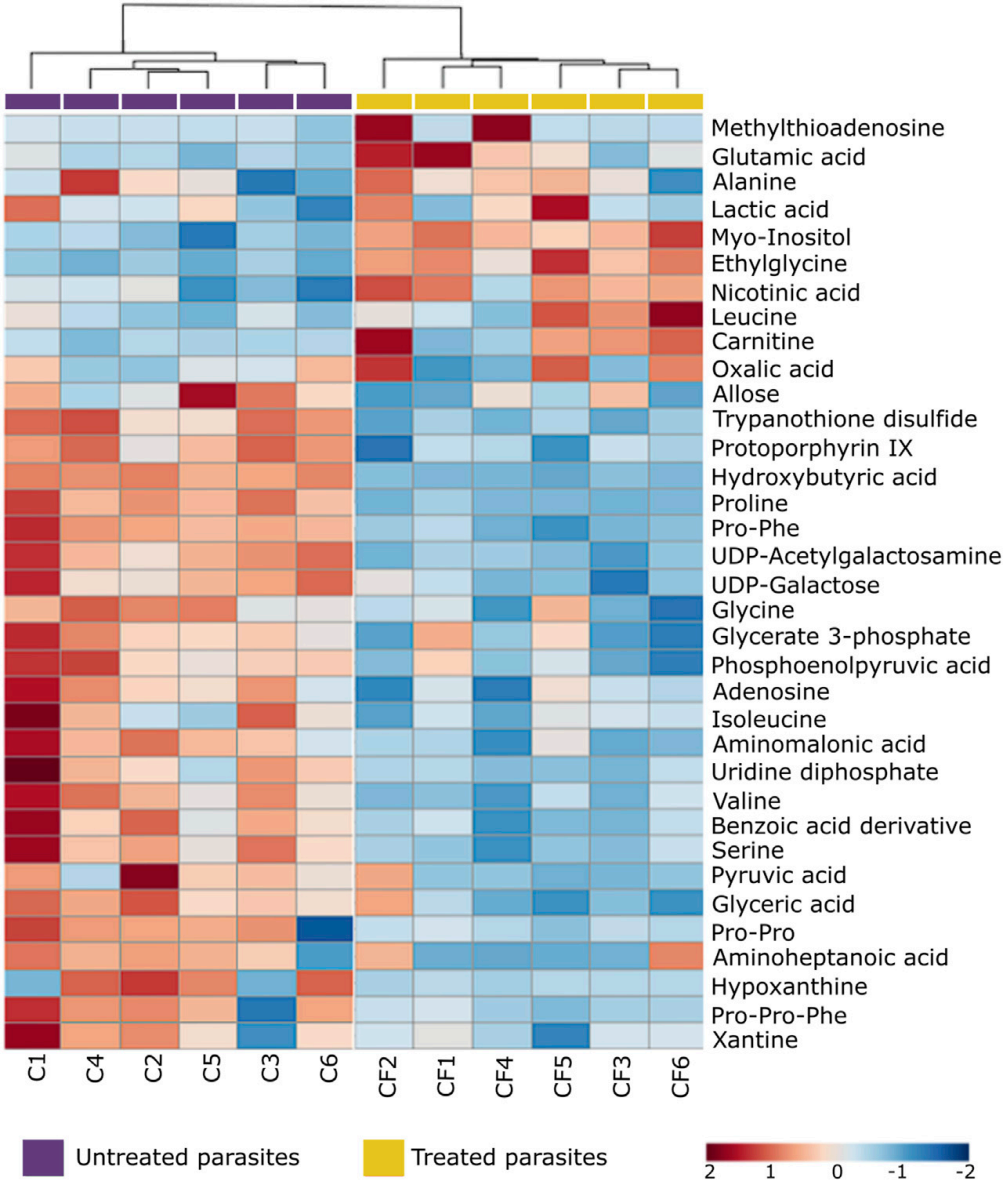
Heat map of non-lipid metabolites with statistically significant variation between *Trypanosoma cruzi* epimastigotes treated with CFHEX and the control group. The columns correspond to each altered metabolite identified, the rows correspond to the analyzed samples divided into the clades: CFHEX-treated parasites (yellow) and untreated parasites (violet). The level of variation is indicated on the right side on a color intensity scale representing relative abundance, where red colors denote metabolite increase and blue colors denote metabolite decrease.

Due to the number of modified metabolites found in parasites treated with CFHEX, it was decided to group them into two large groups. Supplementary Tables S1 and S2 summarize the lipidic and non-lipidic metabolites identified in the treated parasites and present information regarding retention times, coefficient of variation of the chromatographic signal in the QC group, statistical parameters for its selection, probable adducts, fold change (FC), and type of confirmation, among others. Besides, the set of altered metabolites between the two groups was analyzed using heat maps that allow the visualization of metabolite patterns changing between the groups. Thus, blue colors indicate decreased metabolite levels and red colors indicate increased metabolites in treated parasites (Figure 4; Figure 5). Besides, lipid metabolism experienced the greatest variation, with the glycerophospholipid, fatty acid (FA) and sphingolipid classes being the largest representatives (Supplementary Table S1). The group of glycerophospholipids was mainly constituted by lysophospholipids (LPL), of which lysophosphatidylcholines (LPC), lysophosphatidylethanolamines (LPE), lysophosphatidylinositols (LPI), lysophosphatidylglycerol (LPG) and lysophosphatidylserine (LPS), were observed to be increased (Figure 4, colored metabolites on the red color scale). The trends found in the FA subclass (fatty amides, and fatty esters (carnitines)), as well as in the sphingolipids subclass (phosphosphingolipids, sphingoid bases, steroidal lipids and glycerolipids) were also found to have an upward trend (Figure 4, metabolites on the red color scale).

On the other hand, non-lipid compounds were mainly grouped into the classes of nucleosides, acids, and oxygenated organic compounds (Supplementary Table S2). Particularly, in the group of nucleosides, metabolites such as uridine diphosphate (UDP), UDP-acetylgalactosamine, UDP-galactose, 5′-methylthioadenosine and adenosine were identified, all of which showed downward trends (Figure 5, metabolites in the blue color scale). Similar trends were also found in amino acids and peptides (proline, glycine, L-valine, L-isoleucine, serine, Pro-Pro, Pro-Phe, and Pro-Pro-Phe), carboxylic acids (pyruvate and phosphoenolpyruvate), oxygenated organic compounds (glycerate 3 phosphate, glyceric acid, and D-alose), and in other metabolites such as trypanothione, protoporphyrin IX, aminopentanoic acid, hydroxyisovaleric acid, hydroxybutyric acid, xanthine, hypoxanthine and L-carnitine (Figure 5, metabolites in blue color scale).

After an individual analysis of the total altered metabolites in the CFHEX-treated parasites, it was found that the greatest upward changes (FC > 3) occurred in the following compounds: lysophosphatidic acid (18:1); lysophosphatidylcholines (22:1, 18:3, 16:1); lysophosphatidylethanolamines (21:0, 20:0, 20:3, 18:1, 18:3, 17:1), phosphatidylethanolamine (37:2); lysophosphatidylglycerol (28:0); lysophosphatidylinositols (20:4, 18:1, 18:3, 17:1) phosphatidylinositol (40:1); lysophosphatidylserines (21:0, 20:2, 20:3), phosphatidylserine (40:4); dodecanoylcarnitine (12:0), tetradecanoylcarnitine (14:0), palmitoylcarnitine (16:0), hydroxyhexadecanoylcarnitine (16:1), hydroxypalmitoleoylcarnitine (16:1; O), oleoylcarnitine (18:1), linoleoylcarnitine (18:2), carboxyheptadecanoylcarnitine (18:1; O_2_); hexadecanoyl sphinganine phosphomyo-inositol (34:0; O_2_), hexadecanoyl eicosaphingenine phosphomyo-inositol (36:1; O_2_); hydroxyeicosadienoic acid (20:2; O), Prostaglandin F2α (20:2; O_2_); cholestane derivatives (27:0; O_5_, 27:0; O_3_), oleoyl glycerol, stearoyl lactic acid, stearoyl glutamic acid, palmitoyl serine, arachidonoyl tyrosine and ethyl glycine.

In contrast, the greatest downward changes (FC < 0.5) were observed in the lipids: lysophosphatidylcholines (22:2, 20:4, 18:2, 14:0, 13:0, 12:0, 11:0), lysophosphatidylethanolamines (22:5, 15:0, 14:0), lysophosphatidylglycerol (18:2, 16:2), phosphatidylglycerols (40:7, 36:4), lysophosphatydilsernines (20:5, 18:2), ethylacryloylcarnitine (5:1); tetradecanoyl sphingenine phosphomyoinositol (31:1; O_2_) and in the non-lipid metabolites L-carnitine, hydroxybutyric acid, hydroxyisovaleric acid, trypanothione, UDP-acetylgalactosamine, proline, and the peptides Pro-Phe and Pro-Pro-Phe.

**FIGURE 6:**
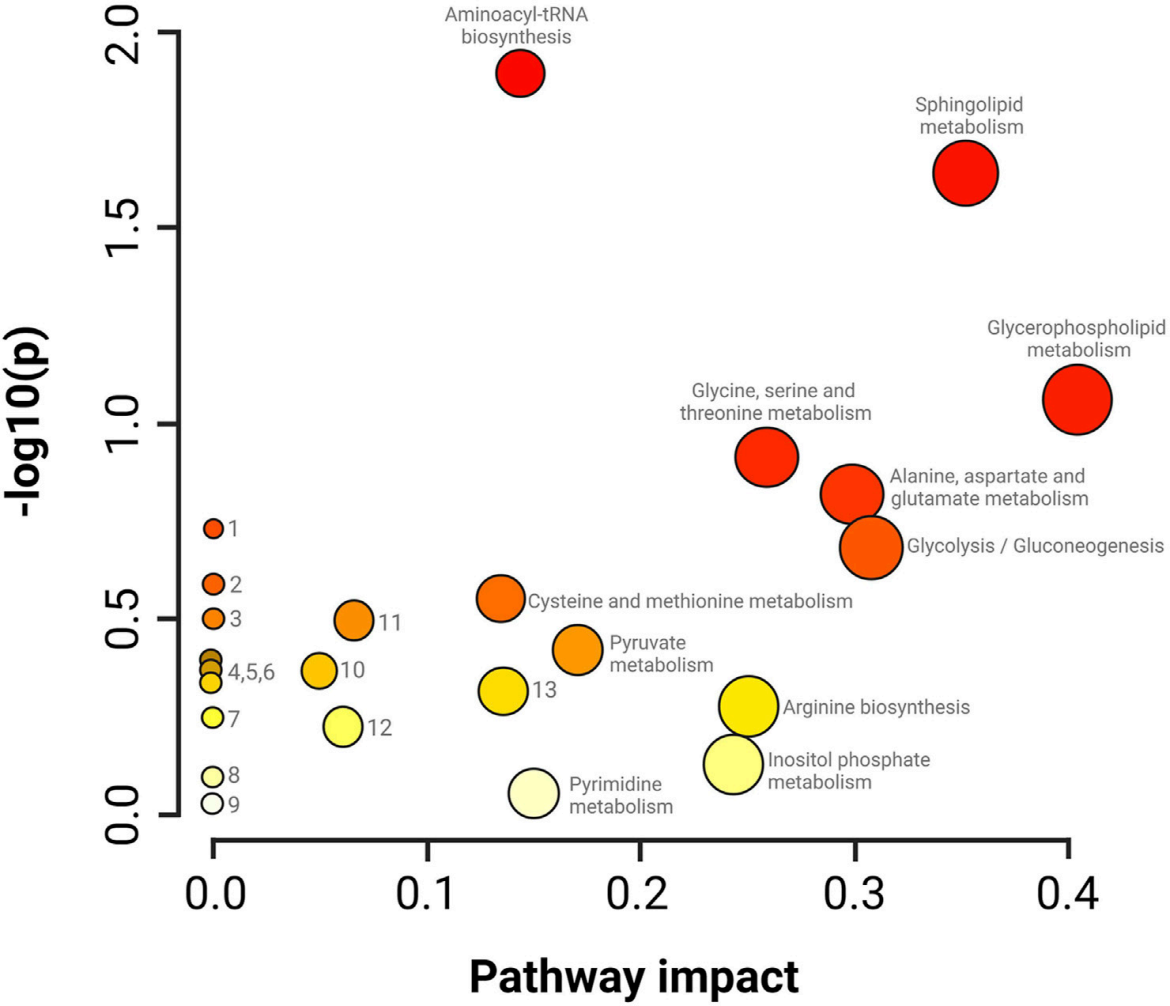
Impact analysis on metabolic pathways. enrichment (y-axis) and topology (x-axis) analysis. 1. Glyoxylate and dicarboxylate metabolism, 2. Taurine and hypotaurine metabolism, 3. Nitrogen metabolism, 4. Porphyrin metabolism, 5. Citrate cycle, 6. Valine, leucine and isoleucine degradation, 7. Nicotinate metabolism and nicotinamide, 8. Metabolism of lipoic acid, 9. Metabolism of aminosugars and sugar nucleotides, 10. Degradation of fatty acids, 11. Metabolism of arginine and proline, 12. Metabolism of purines, 13. Metabolism of glutathione. The darker colors indicate a greater number of changes in the metabolic pathway, while the size of the circle corresponds to the impact on the pathway according to the importance of the altered metabolite.

The summary of the main routes affected in the treated parasites is presented in Figure 6. The results of the pathway enrichment analysis with the topology analysis show that while the metabolic pathways with the greatest displacement in the Y-axis, denoted in dark colors, indicate the greatest changes in the metabolic pathway according to the number of metabolites participating in each affected pathway, the displacement on the X-axis, related to the size of the circle, indicates the impact on the metabolic pathway according to the importance of the altered metabolite, measured according to the number of connections that it presents on the pathway (Figure 6) (Xia et al., 2011). The metabolic pathways that were observed to be the most affected by CFHEX treatment (denoted in the red color scale and larger circles) were related to amino acid metabolism, which was found to be preferentially decreased. Thus, the biosynthesis of transfer RNA stands out due to the alteration of glycine, serine, valine, alanine, isoleucine, leucine, proline, and glutamate aminoacyl-tRNAs. Changes in the metabolism of glycine, serine, threonine, alanine, aspartate, glutamate, cysteine, and methionine were also observed as well as on the arginine biosynthesis. Another altered pathway was glycolysis caused by the decrease of phosphoenolpyruvate, glycerate 3-phosphate, and pyruvate. Finally, the metabolism of sphingolipids and glycerophospholipids was also affected by the overproduction of sphingosine 1 phosphate, sphingosine, sphinganine, phosphatidic acid, phosphatidylcholines and phosphatidylethanolamines.

**FIGURE 7:**
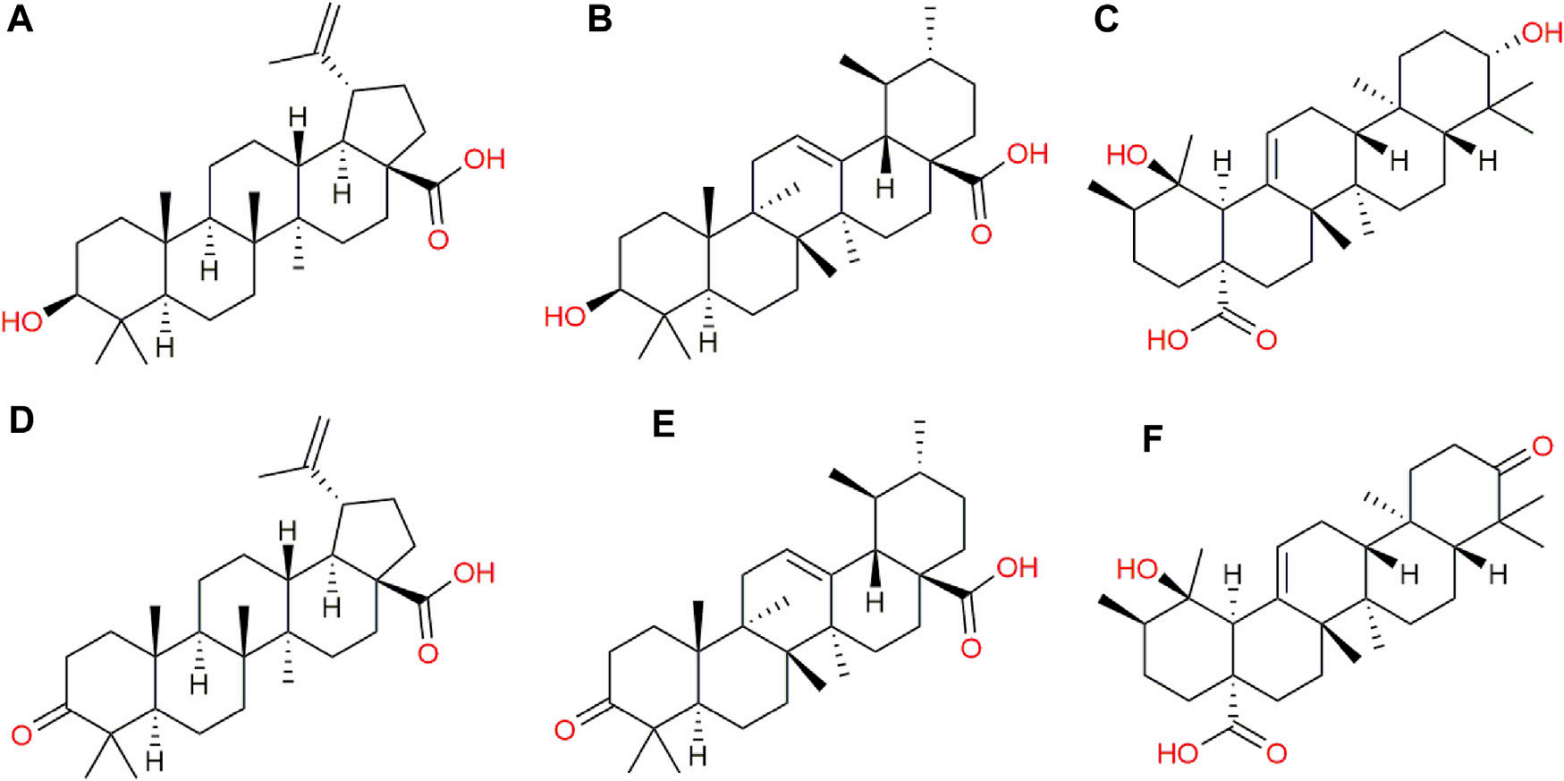
Exogenous metabolites found in epimastigotes treated with CFHEX extract. **(A)** Betulinic acid, **(B)** Ursolic acid, **(C)** Pomolic acid, **(D)** Betulonic acid, **(E)** Ursonic acid, **(F)** Pomonic acid.

In addition to the metabolomic alterations described in the treated parasites, a series of compounds exogenous to *T. cruzi* were detected and found exclusively in the treated parasites and those would be constituents of the CFHEX extract (Pardo-Rodriguez et al., 2022). These compounds were: betulinic acid, ursolic acid, pomolic acid and their oxidized forms, betulonic acid, ursonic acid and pomonic acid. All these compounds are classified as pentacyclic triterpenes (compounds with a skeleton of 30 carbons that form five cycles) that are widely distributed in plant families and had been previously reported in *C. fimbriata* (Pardo-Rodriguez et al., 2022) (Figure 7).

## 4 Discussion

*T. cruzi* is a protozoan with a complex life cycle that involves vertebrate and invertebrate hosts and extracellular and intracellular stages (Rassi et al., 2010), exposing it, among other factors, to various sources of carbon as glucose and lipids in the mammalian host and amino acids, mainly proline in the insect vector (Cazzulo et al., 1985; Cazzulo, 1992; Michels et al., 2021). In particular, the epimastigote stage can use multiple carbon sources, however, in culture media it preferentially uses carbohydrates as a substrate for energy metabolism during the exponential phase and amino acids during the stationary phase (Cazzulo et al., 1985; Cazzulo, 1992; Silber et al., 2002; Barisón et al., 2017).

Glucose metabolism occurs in a similar way to other trypanosomatids, part of the enzymes of the glycolytic pathway in *T. cruzi* are compartmentalized within organelles of peroxisomal origin called glycosomes (Van Hellemond et al., 2005; Quiñones et al., 2020; Michels et al., 2021). Additionally, *T. cruzi* has a mitochondrial system that includes enzymes of the tricarboxylic acid cycle (TAC), as well as mitochondrial electron transport chain complexes (Stoppani et al., 1980; César Carranza et al., 2009), present at all stages of the parasite’s life cycle. The glycolytic pathway is organized such that the first seven enzymes that catabolize glucose to glycerate 3-phosphate (G3P) are found within glycosomes, while the last three enzymes in the pathway reside in the cytosol (Bringaud et al., 2006; Michels et al., 2021), with the latter three enzymes being phosphoglycerate mutase, enolase and pyruvate kinase responsible for the transformation of G3P, phosphoenolpyruvate (PEP) and pyruvate (PYR), respectively.

G3P, PEP and PIR were found decreased after epimastigotes-CFHEX treatment, which may suggest energetic alterations in the treated parasites, as well as effects on metabolic pathways that use these substrates or derivatives of them. On the other hand, exposure of epimastigotes to CFHEX extract also reduced the levels of some nucleosides, which in normal conditions contribute to energy generation. The pathway that allows the formation of UDP-galactose is linked to the generation of glucose 6-phosphate and this last metabolite could be incorporated into the glycolytic pathway to generate ATP and feed other metabolic pathways such as pentose phosphate (Roper and Ferguson, 2003). Other biosynthetic pathways that participate as carbon and energy sources through the generation of intermediates for the TAC, such as the amino acids proline (Silber et al., 2002; Silber et al., 2005; Martins et al., 2009; Paes et al., 2013), valine, isoleucine, and serine (Hamptont, 1971; Sylvester and Krassner, 1976; Cazzulo, 1994; Silber et al., 2005; Manchola et al., 2016), were also found downward trends.

The effects observed on processes related to carbohydrate and amino acid-dependent energy production in epimastigotes are like those reported after nutritional stress induced by long periods of starvation. Souza et al. evaluated the role of FA oxidation after depriving *T. cruzi* epimastigotes cultures of glucose, finding that, in the absence of glucose, lipid droplets become the main sources of FA, which help the body survive nutritional stress by producing acetyl-CoA that fuels TAC, contributing to mitochondrial ATP production (Souza et al., 2021). In summary, epimastigotes use FA as carbon and energy source when glucose and amino acids are not available. In this context, the use of lipids as a source of carbon and energy in the treated parasites is suggested by the findings that the family of lipids conjugated to carnitine presented upward trends.

Interestingly, carnitines were the family of compounds that presented the greatest alteration in the study. Acyl-carnitines have the biological function of transporting FA into the mitochondria to be substrates for β-oxidation. To do this, carnitines form conjugates with the FA that will be oxidized, generating NADH, FADH_2_ and acetyl CoA in each round (Longo et al., 2016). Both NADH and FADH_2_ enter the electron transport chain to produce ATP (Carracedo et al., 2013). On the other hand, the acetyl CoA generated in the oxidation processes can feed the production of TAC intermediates or be part of new lipid synthesis (Ginger et al., 2000; Carracedo et al., 2013). However, it cannot be ruled out that the accumulation of carnitines is due to a malfunction of mitochondrial activity that prevents the catabolism of this type of metabolite.

FA can be acquired in three ways: exogenous FA that enter cells; FA that arise through *de novo* synthesis from acetyl-coA; and fatty acids that are released within the cell by hydrolysis of acylated proteins, phospholipids, and triglycerides (Koundouros and Poulogiannis, 2020). The lipid composition found in *T. cruzi* varies according to the stage analyzed; however, they are mainly represented by triacylglycerides, phosphatidylcholines, phosphatidylethanolamines and phosphatidylinositols (Cunha-E-Silva et al., 2002; Booth and Smith, 2020). These types of compounds incorporated the greatest global changes in the metabolism of the treated parasites, with a large part of these showing upward trends. Of these, the group of LPL had the largest number of representatives. LPL are metabolic intermediates normally generated through the active hydrolyzation of phospholipases. These enzymes cleave intracellular phospholipids from the cell membrane, generating a variety of products such as LPL, FA, diacylglycerols, phosphocholine, phosphoinositides and phosphatidic acid, among others (Belaunzarán et al., 2011). All these intermediate metabolites can contribute to the generation of ATP through oxidation to acetyl-coA and then be incorporated into TAC. However, the role of this mechanism in energy generation during nutritional stress events is still unknown. Other studies carried out in pathologies in which there is an increase in reactive oxygen species (ROS), such as diabetes and obesity, have associated the selective loss of a glycerophospholipid fatty acyl residue with the overproduction of ROS through lipid peroxidation. However, this mechanism is still under study (Reis and Spickett, 2012; Fuchs, 2014; Chen et al., 2018; Engel et al., 2021).

The use of glycerophospholipids as a substrate to produce intermediates such as LPL can affect the composition of biological membranes and, consequently, their selective permeability, leading to cell lysis. Importantly, the accumulation of this type of lipid in the treated parasites entails toxic effects, since high concentrations alter the structure of the membrane and cause cell lysis. Finally, some research suggests that FA oxidation may be a permanent source of reactive oxygen species, which can cause endoplasmic reticulum stress and changes in mitochondrial membrane potentials, causing apoptotic death (Bowes et al., 1993; Ly et al., 2017; Tan et al., 2020).

Although the observed alterations have been related to a possible mechanism of energy alteration, the increases or deficiencies of some metabolites suggest involvement in other biological processes. Changes in the content of amino acids such as proline, glutamate, serine, glycine, and leucine can affect protein synthesis, resistance to nutritional, osmotic, thermal, and oxidative stress, as well as invasion, replication, and metacyclogenesis processes, among others (Contreras et al., 1985; Pereira et al., 2002; Silber et al., 2005; Magdaleno et al., 2011; Paes et al., 2013; Marchese et al., 2018). On the other hand, alterations in nucleosides would directly affect the construction of macromolecules such as glycoconjugates, DNA and RNA, which would affect the invasion and processes dependent on nucleic acids, such as replication, transcription and protein synthesis (Landfear et al., 2004; MacRae et al., 2006). Finally, both energy imbalances and low trypanothione levels, observed in parasites treated with CFHEX, may suggest an increase in reactive oxygen species (ROS) as well as imbalances in redox potentials (Fairlamb et al., 1985; Bond et al., 1999; Schönfeld et al., 2010; Menna-Barreto and De Castro, 2014). ROS can oxidize macromolecules such as lipids, nucleic acids, and proteins. Besides, it can cause endoplasmic reticulum stress and mitochondrial damage, alterations that can promote, among many other effects, programmed cell death (Jarzab and Stryjecka-Zimmer, 2008).

Previously, the analysis of the mechanisms of death induced by the CFHEX extract in epimastigotes and trypomastigotes of *T. cruzi* evidenced a trypanosomicidal mechanism associated with programmed cell death. The present investigation found a series of highly expressed proapoptotic compounds such as sphingosine, sphingosine 1 phosphate and ceramides (Cuvillier, 2002; Davaille et al., 2002; Koeller and Heise, 2011), which support the effects of programmed cell death previously evidenced (Pardo-Rodriguez et al., 2022). The trypanosomicidal effects can be related to the presence of exogenous terpenes found in the parasites and previously described in *C. fimbriata* (Pardo-Rodriguez et al., 2022). Ursolic acid induced a significant reduction in amastigotes in RAW macrophage cultures infected with trypomastigotes compared with the untreated cultures (Vanrell et al., 2020). Other studies conducted in models of acute infection in BALB/c albino male mice found that oral treatment with ursolic acid at a concentration of 20 mg/kg/day reduced parasitemia, measured at the parasitemic peak, after infection with trypomastigotes of strain Y by 60% (Da Silva Ferreira et al., 2013). On the other hand, betulinic acid inhibits cellular populations of all three stages of *T. cruzi* without causing toxicity in LLC-MK2 cells at the concentrations used. (200-1,600 μM). Furthermore, the treated parasites displayed alterations in cell membrane integrity, mitochondrial membrane potential, and reservosome inflammation, along with an increased production of reactive oxygen species (Sousa et al., 2017).

**FIGURE 8:**
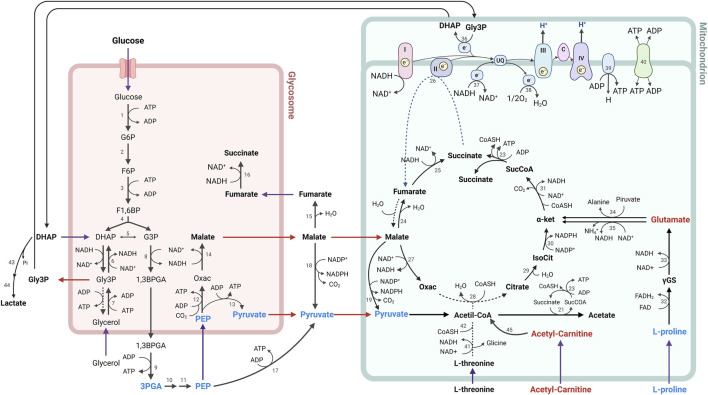
Metabolic processes for energy generation in *Trypanosoma cruzi*. The metabolites found in lower concentration are represented in blue, while the metabolites found in higher concentration in parasites treated with CFHEX compared to untreated parasites are represented in red. Numbers indicate participating enzymes: 1, hexokinase; 2, glucose-6-phosphate isomerase; 3, phosphofructokinase; 4, aldolase; 5, triose-phosphate isomerase; 6, glycerol-3-phosphate dehydrogenase; 7, glycerol kinase; 8, glyceraldehyde-3-phosphate dehydrogenase; 9, phosphoglycerate kinase B; 10, phosphoglycerate mutase; 11, enolase; 12, phosphoenolpyruvate carboxykinase; 13, pyruvate phosphate dikinase; 14, glycosomal malate dehydrogenase; 15, cytosolic fumarase; 16, glycosomal NADH-dependent fumarate reductase, 17, pyruvate kinase; 18, cytosolic malic enzyme; 19, mitochondrial malic enzyme; 20, pyruvate dehydrogenase complex; 21, acetate:succinate CoA-transferase; 22, acetyl-CoA thioesterase; 23, succinyl-CoA synthetase; 24, mitochondrial fumarase; 25, mitochondrial NADH-dependent fumarate reductase; 26, succinate dehydrogenase (respiratory chain complex II); 27, mitochondrial malate dehydrogenase; 28, citrate synthase; 29, aconitase; 30, isocitrate dehydrogenase; 31, α-ketoglutarate dehydrogenase; 32, L-proline dehydrogenase; 33, pyrroline-5-carboxylate dehydrogenase; 34, alanine aminotransferase; 35, glutamate dehydrogenase; 36, FAD-dependent mitochondrial glycerol-3-phosphate dehydrogenase; 37, rotenone-insensitive NADH dehydrogenase; 38, alternative oxidase; 39, FoF1-ATP synthase; 40, ADP/ATP carrier; 41, L-threonine dehydrogenase; 42, AKCT, 2-amino-3-ketobutyrate CoA-transferase; 43, methylglyoxal reductase; 44, lactaldehyde dehydrogenase; 45, Fatty acid β-oxidation; I, III, IV, respiratory chain complexes.

In summary, the metabolic alterations observed in parasites treated with CFHEX may reflect energetic alterations associated with glucose, hexoses and some amino acids which suggests that the energy demands of the parasite could be supplied from the β-oxidation of FA and the production of TAC intermediates (Figure 8). Although, with the results obtained here, it is not possible to specify the mechanism by which the alteration of energy metabolism is taking place, investigations carried out with betulinic, ursolic and oleanolic acids have shown a deprivation of glycolytic metabolism in cancer cells. In these studies, the glycolytic decline is associated with signaling pathways such as mTOR, and AKT, which modulate the expression of glycolytic pathway enzymes such as hexokinase, phosphofructokinase, and pyruvate kinase. Additionally, it has been observed that apoptotic processes induced by oxidative stress are favored in cells treated with ursolic, betulinic and pomolic acids (Liu et al., 2014; Lewinska et al., 2017; Zheng et al., 2019; Wang et al., 2021). Other studies have found inhibitory effects in trypanosomatid species after exposure to betulinic and ursolic acids associated with alterations in the mitochondrial membrane potential and increases in oxidative stress, which in turn implies effects on oxidative phosphorylation (Yamamoto et al., 2015; Bossolani et al., 2017; Sousa et al., 2017; Albuquerque et al., 2020).

With this panorama, two possible hypotheses arise as an explanation for glucose deprivation or depletion. The triterpenes that constitute CFHEX (betulinic, ursolic and pomolic acid), which were found inside the treated parasites, can inhibit the uptake of glucose or its metabolization by modulating signaling pathways (Liu et al., 2014; Lewinska et al., 2017; Zheng et al., 2019; Wang et al., 2021). Likewise, and although it is an approach little studied, the possible inhibition exerted by triterpenes on glucose transporters and enzymes of the glycolytic pathway of *T. cruzi* cannot be ruled out. This last point becomes relevant when comparing the identities of the transporters and enzymes present in trypanosomatids with respect to those found in humans. In summary, the inhibition of glycolysis would induce the consumption of other carbon sources such as amino acids and lipids, which would serve as generators of TAC intermediates, that would directly feed mitochondrial oxidative phosphorylation, making the treated parasites dependent on this last mechanism to meet energy demands.

However, a second mechanism has been proposed for certain triterpenes, and it has been found that those compounds could modify the potentials of mitochondrial membranes (Ψm) (Hordyjewska et al., 2019), which, in turn, may impact electron transport and oxidative phosphorylation. These findings may suggest that the triterpenes found as major compounds in CFHEX have the ability to uncouple mitochondrial function and promote metabolic changes that induce a death similar to apoptosis in the treated parasites.

Collectively, our results showed that the hexanic extract of *C. fimbriata* induces alterations in energy metabolism in *T. cruzi* epimastigotes that are compatible with CFHEX induced apoptosis-like death. The finding of pentacyclic triterpenes in the metabolome of *C. fimbriata* treated parasites renders this extract as a novel source of triterpene compounds, which in the future may contribute to new alternatives for the control of *T. cruzi* infection. To our knowledge, this is the first investigation where the general metabolic context of *T. cruzi* correlated to the exposure of extracts rich in pentacyclic triterpenes. The metabolomic analyses performed on the epimastigote stage reflect the changes generated generated from the CFHEX treatment, however, they would only indicate the steady states of the metabolites at the selected treatment time (36 h). Therefore, the discussion was carried out by correlating the states of the altered metabolites with their biological functions. It is necessary for future studies to establish the metabolic pathway fluxes involved in the inhibition mechanisms induced in the *T. cruzi* stages by the CFHEX extract.

## 5 Conclusion

The effect of the hexanic extract of *C. fimbriata* on the metabolism of *T. cruzi* epimastigotes is related to multiple biosynthetic pathways, among which those associated with energy metabolism stand out, which in turn is connected to the induction of apoptosis-like observed in treated parasites. The constituent triterpenes of CFHEX such as betulinic and ursolic acids were found inside the treated parasites and may be related to the trypanosomicidal effects together with the metabolomic modifications.

## Figures and Tables

**Table:** 

**CAR**	Carnitine
**CER**	Ceramide
**CFHEX**	C. fimbriata hexanic extract
**GC-MS**	Gas chromatography coupled to mass spectrometry
**EtOH**	Ethanol
**FA**	fatty acid
**FADH2**	Flavin adenine dinucleotide
**FBS**	Fetal Bovine Serum
**FC**	Fold change
**PEP**	Phosphoenolpyruvate
**G3P**	Glycerate 3 phosphate
**HILIC**	Hydrophilic Interaction Liquid Chromatography
**IC90**	90% Inhibitory Concentration
**SL**	Steroidal lipid
**LC-MS**	Liquid chromatography coupled to mass spectrometry
**LIT**	Liver Infusion Tryptose
**LPC**	Lysophosphatidylcholines
**LPE**	Lysophosphatidylethanolamines
**LPG**	Lysophosphatidylglycerol
**LPI**	Lysophosphatidylinositols
**LPL**	Lysophospholipids
**LPS**	Lysophosphatidylserine
**MeOH**	Methanol
**MG**	Monoglyceride
**NADH**	Nicotine Adenine Dinucleotide
**OPLS-DA**	Orthogonal Partial Least Squares with Discriminant Analysis
**PCA**	Principal Component Analysis
**PIR**	Pyruvate
**PLS**	Partial Least Squares
**QC**	Quality Control
**TAC**	Tricarboxylic Acid Cycle
**ROS**	Reactive oxygen species
**TR**	Retention Time
**UDP**	Uridine Diphosphate
**VIP**	Importance of the variable in the projection

## Data Availability

The raw data supporting the conclusions of this article will be made available by the authors, without undue reservation.
