# Supplementary material for: Clethra fimbriata hexanic extract triggers alteration in the energy metabolism in epimastigotes of Trypanosoma cruzi
Source: Front Mol Biosci. 2023 Sep 25;10:1206074. doi: 10.3389/fmolb.2023.1206074 (PMC10561390; doi:10.3389/fmolb.2023.1206074)
Supplement: Supplementary file 1 [file Table1.DOCX]

**Supplementary Table 1.** Compounds of a lipid nature that presented statistically significant differences between treated and untreated parasites, identified by untargeted metabolomics using the four analytical platforms.

| **R.T.** | **% CV QC** | **F.C.** | ***P value*** | **VIP** | **Add.** | **M.E.** | **M.W.** | **ID.** | **Chem. Form.** | **ID.Level*** | **Plat.** |
| --- | --- | --- | --- | --- | --- | --- | --- | --- | --- | --- | --- |
| ***Glycerophospholipids*** | | | | | | | | | | | |
| *Glycerophosphates* | | | | | | | | | | | |
| 27.02 | 10.0 | 4.01 | 0.002 | 1.74 | [M-H]^-^ | 1 | 436.259 | LPA18:1 | C_21_H_41_O_7_P | 2 | LC- |
| 19.72 | 3.6 | 0.27 | 0.002 | - | [M-H]^-^ | 1 | 434.2433 | LPA18:2 | C_21_H_39_O_7_P | 2 | LC- |
| *Glycerophosphocholines* | | | | | | | | | | | |
| 23.41 | 8.9 | 1.64 | 0.004 | 3.87 | [M+HCOOH-H]^-^ | 1 | 521.3481 | LPC18:1 | C_26_H_52_NO_7_P | 2 | LC± |
| 18.53 | 5.5 | 17.46 | 0.002 | 2.70 | [M+HCOOH-H]^-^ | 1 | 517.3168 | LPC18:3 | C_26_H_48_NO_7_P | 2 | LC± |
| 25.86 | 18.7 | 1.91 | 0.004 | 1.20 | [M+HCOOH-H]^-^ | 1 | 549.3794 | LPC20:1 | C_28_H_56_NO_7_P | 2 | LC± |
| 13.87 | 4.0 | 0.17 | 0.002 | - | [M-H]^-^ | 0 | 425.2542 | LPC11:0 | C_19_H_40_NO_7_P | 3 | LC- |
| 13.87 | 5.8 | 0.17 | 0.002 | - | [M+HCOOH-H]^-^ | 0 | 439.2699 | LPC12:0 | C_20_H_42_NO_7_P | 2 | LC± |
| 20.83 | 4.4 | 0.35 | 0.002 | - | [M+HCOOH-H]^-^ | 1 | 543.3325 | LPC20:4 | C_28_H_50_NO_7_P | 2 | LC± |
| 17.87 | 7.6 | 0.47 | 0.004 | - | [M-H]^-^ | 1 | 453.2855 | LPC13:0 | C_21_H_44_NO_7_P | 3 | LC- |
| 21.22 | 10.2 | 4.00 | 0.002 | - | [M-H]^-^ | 0 | 493.3168 | LPC16:1 | C_24_H_48_NO_8_P | 3 | LC- |
| 17.87 | 6.9 | 0.48 | 0.004 | - | [M+HCOOH-H]^-^ | 1 | 467.3012 | LPC14:0 | C_22_H_46_NO_7_P | 2 | LC± |
| 19.97 | 3.5 | 2.23 | 0.004 | 1.24 | [M+H]^+^ | 5 | 481.3168 | LPC15:0 | C_23_H_48_NO_7_P | 3 | LC+ |
| 22.33 | 8.5 | 0.71 | 0.041 | - | [M+Cl]^-^ | 1 | 545.3481 | LPC20:3 | C_28_H_52_NO_7_P | 3 | LC- |
| 25.46 | 11.7 | 1.47 | 0.041 | - | [M+HCOOH-H]^-^ | 1 | 535.3638 | LPC19:1 | C_27_H_54_NO_7_P | 2 | LC± |
| 20.68 | 5.4 | 1.18 | 0.009 | 1.21 | [M+H]^+^ | 0 | 541.3168 | LPC20:5 | C_28_H_48_NO_7_P | 2 | LC+ |
| 21.22 | 11.7 | 2.75 | 0.002 | 1.46 | [M+H]^+^ | 5 | 507.3325 | LPC17:1 | C_25_H_50_NO_7_P | 2 | LC+ |
| 21.4 | 8.5 | 0.42 | 0.002 | 1.64 | [M+K]^+^ | 3 | 519.3325 | LPC18:2 | C_26_H_50_NO_7_P | 3 | LC+ |
| 25.29 | 7.4 | 0.25 | 0.002 | 1.50 | [M+Na]^+^ | 9 | 575.3951 | LPC22:2 | C_30_H_58_NO_7_P | 3 | LC+ |
| 26.99 | 8.7 | 1.94 | 0.002 | 1.28 | [M+Na]^+^ | 10 | 551.3951 | LPC20:0 | C_28_H_58_NO_7_P | 3 | LC+ |
| 32.8 | 10.0 | 3.56 | 0.002 | 1.61 | [M+H]^+^ | 6 | 577.4107 | LPC22:1 | C_30_H_60_NO_7_P | 3 | LC+ |
| 9.47 | 2.2 | 2.14 | 0.004 | 1.24 | [M+Cl]^-^ | 10 | 729.5309 | PC32:2 | C_40_H_76_NO_8_P | 2 | HILIC- |
| *Glycerophosphoethanolamine* | | | | | | | | | | | |
| 20.46 | 9.0 | 0.59 | 0.002 | 3.92 | [M-H]^-^ | 3 | 477.2855 | LPE18:2 | C_23_H_44_NO_7_P | 2 | LC± [HILIC-] |
| 23.92 | 9.8 | 3.17 | 0.002 | 3.36 | [M-H]^-^ | 0 | 479.3012 | LPE18:1 | C_23_H_46_NO_7_P | 2 | LC± [HILIC-] |
| 18.34 | 8.8 | 10.06 | 0.002 | 1.31 | [M-H]^-^ | 1 | 475.2699 | LPE18:3 | C_23_H_42_NO_7_P | 2 | LC± |
| 13.87 | 5.8 | 0.17 | 0.002 | - | [M+HCOOH-H]^-^ | 0 | 439.2699 | LPE15:0 | C_20_H_42_NO_7_P | 3 | LC- |
| 13.87 | 4.0 | 0.17 | 0.002 | - | [M-H]^-^ | 0 | 425.2542 | LPE14:0 | C_19_H_40_NO_7_P | 3 | LC- |
| 16.74 | 9.6 | 0.34 | 0.002 | - | [M-H]^-^ | 0 | 449.2542 | LPE16:2 | C_21_H_40_NO_7_P | 2 | LC- |
| 18.56 | 11.7 | 0.52 | 0.002 | - | [M-H]^-^ | 0 | 463.2699 | LPE17:2 | C_22_H_42_NO_7_P | 2 | LC± |
| 18.94 | 8.9 | 0.69 | 0.002 | - | [M-H]^-^ | 0 | 451.2699 | LPE16:1 | C_21_H_42_NO_7_P | 2 | LC± |
| 18.66 | 1.3 | 0.65 | 0.015 | - | [M-H]^-^ | 0 | 453.2855 | LPE16:0 | C_21_H_44_NO_7_P | 3 | LC- |
| 27.84 | 14.3 | 2.45 | 0.002 | - | [M-H]^-^ | 1 | 535.3638 | LPE22:2 | C_27_H_54_NO_7_P | 3 | LC- |
| 18.53 | 9.6 | 15.24 | 0.002 | 1.41 | [M-H]^-^ | 1 | 503.3012 | LPE20:3 | C_25_H_46_NO_7_P | 3 | LC- |
| 20.99 | 9.2 | 4.12 | 0.002 | - | [M-H]^-^ | 0 | 465.2855 | LPE17:1 | C_22_H_44_NO_7_P | 3 | LC± |
| 21.16 | 10.3 | 0.42 | 0.004 | - | [M-H]^-^ | 2 | 525.2855 | LPE22:6 | C_27_H_44_NO_7_P | 2 | LC± |
| 23.41 | 8.3 | 1.64 | 0.004 | 1.69 | [M-H]^-^ | 0 | 507.3325 | LPE20:1 | C_25_H_50_NO_7_P | 3 | LC- |
| 22.8 | 16.9 | 0.50 | 0.004 | - | [M-H]^-^ | 1 | 527.3012 | LPE22:5 | C_27_H_46_NO_7_P | 2 | LC± |
| 28.73 | 16.8 | 1.78 | 0.041 | - | [M-H]^-^ | 1 | 535.3638 | LPE22:1 | C_27_H_54_NO_7_P | 3 | LC- |
| 27.87 | 10.8 | 3.31 | 0.002 | 1.16 | [M+HCOOH-H]^-^ | 1 | 523.3638 | LPE21:0 | C_25_H_52_NO_7_P | 2 | LC± |
| 24.47 | 3.4 | 3.70 | 0.002 | 1.47 | [M+H]^+^ | 5 | 509.3481 | LPE20:0 | C_25_H_52_NO_7_P | 3 | LC± |
| 8.81 | 2.9 | 2.93 | 0.009 | 1.01 | [M-H]^-^ | 1 | 697.5046 | PEO-34:4 | C_39_H_72_NO_7_P | 2 | HILIC- |
| 9.35 | 9.7 | 3.93 | 0.002 | 1.10 | [M+Cl]^-^ | 9 | 757.5622 | PE37:2 | C_42_H_80_NO_8_P | 2 | HILIC- |
| *Glycerophosphoglycerols* | | | | | | | | | | | |
| 1.56 | 3.3 | 16.53 | 0.002 | 1.08 | [M-H]^-^ | 4 | 666.4472 | PG28:0 | C_34_H_67_O_10_P | 3 | HILIC- |
| 1.66 | 4.3 | 8.64 | 0.002 | 1.06 | [M-H]^-^ | 4 | 802.5724 | PG38:2 | C_44_H_83_O_10_P | 3 | HILIC- |
| 4.98 | 5.6 | 0.13 | 0.002 | 1.20 | [M-H]^-^ | 2 | 820.5254 | PG40:7 | C_46_H_77_O_10_P | 3 | HILIC- |
| 5.23 | 4.0 | 0.09 | 0.002 | 1.44 | [M-H]^-^ | 1 | 770.5098 | PG36:4 | C_42_H_75_O_10_P | 3 | HILIC- |
| 7.05 | 9.2 | 0.08 | 0.002 | 1.52 | [M-H]^-^ | 1 | 508.2801 | LPG18:2 | C_24_H_45_O_9_P | 2 | HILIC- |
| 10.46 | 3.1 | 2.41 | 0.002 | 1.31 | [M+Cl]^-^ | 5 | 564.3427 | LPG22:2 | C_28_H_53_O_9_P | 3 | HILIC- |
| *Glycerophosphoinositols* | | | | | | | | | | | |
| 21.65 | 5.3 | 6.05 | 0.002 | 8.06 | [M-H]^-^ | 2 | 598.3118 | LPI18:1 | C_27_H_51_O_12_P | 2 | LC± [HILIC-] |
| 16.9 | 1.8 | 14.57 | 0.002 | 1.63 | [M-H]^-^ | 1 | 594.2805 | LPI18:3 | C_27_H_47_O_12_P | 2 | LC- [HILIC-] |
| 19.42 | 6.1 | 9.03 | 0.002 | 1.44 | [M-H]^-^ | 1 | 584.2962 | LPI17:1 | C_26_H_49_O_12_P | 2 | LC- [HILIC-] |
| 21.71 | 16.3 | 4.56 | 0.002 | 1.09 | [M+HCOOH-H]^-^ | 5 | 620.2962 | LPI20:4 | C_29_H_49_O_12_P | 2 | LC- |
| 19.31 | 4.2 | 2.09 | 0.002 | - | [M-H]^-^ | 0 | 644.2962 | LPI22:6 | C_31_H_49_O_12_P | 3 | LC- |
| 17.39 | 4.9 | 2.21 | 0.004 | - | [M-H]^-^ | 0 | 570.2805 | LPI16:1 | C_25_H_47_O_12_P | 2 | LC- |
| 17.07 | 7.8 | 1.50 | 0.041 | - | [M-H]^-^ | 0 | 582.2805 | LPI17:2 | C_26_H_47_O_12_P | 2 | LC- |
| 26.7 | 7.3 | 1.58 | 0.041 | - | [M-H]^-^ | 1 | 600.3275 | LPI18:0 | C_27_H_53_O_12_P | 2 | LC- [HILIC-] |
| 23.13 | 19.6 | 7.71 | 0.002 | 1.59 | [M+Na]^+^ | 7 | 920.6354 | PI40:1 | C_49_H_93_O_13_P | 3 | LC+ |
| *Glycerophosphoserines* | | | | | | | | | | | |
| 26.91 | 14.9 | 4.00 | 0.002 | - | [M+H]^+^ | 0 | 523.3638 | LPS21:0 | C_25_H_52_NO_7_P | 3 | LC+ |
| 19.7 | 10.1 | 0.22 | 0.002 | 1.69 | [M-H]^-^ | 0 | 521.2754 | LPS18:2 | C_24_H_44_NO_9_P | 2 | LC± |
| 19.7 | 4.4 | 0.28 | 0.002 | - | [M-H]^-^ | 5 | 543.2597 | LPS20:5 | C_26_H_42_NO_9_P | 3 | LC- |
| 23.65 | 18.6 | 3.00 | 0.002 | - | [M-H-H_2_O]^-^ | 4 | 549.3067 | LPS20:2 | C_26_H_48_NO_9_P | 4 | LC- |
| 23.15 | 18.1 | 3.23 | 0.002 | - | [M-H]^-^ | 4 | 547.291 | LPS20:3 | C_26_H_46_NO_9_P | 3 | LC- |
| 21.16 | 6.6 | 0.73 | 0.004 | - | [M-H]^-^ | 3 | 545.2754 | LPS20:4 | C_26_H_44_NO_9_P | 2 | LC- |
| 23.9 | 6.1 | 1.90 | 0.004 | - | [M+HCOOH-H]^-^ | 5 | 569.2754 | LPS22:6 | C_28_H_44_NO_9_P | 3 | LC- |
| 10.77 | 4.5 | 2.36 | 0.009 | 1.13 | [M-H]^-^ | 5 | 845.6146 | PS40:1 | C_46_H_88_NO_10_P | 3 | HILIC- |
| 10.82 | 2.2 | 5.55 | 0.002 | 1.42 | [M-H]^-^ | 2 | 839.5676 | PS40:4 | C4_6_H_82_NO_10_P | 2 | HILIC- |
| ***Fatty acids*** | | | | | | | | | | | |
| *Fatty acids and derivatives* | | | | | | | | | | | |
| 23.19 | 11.7 | 2.11 | 0.002 | 1.44 | [M-H]^-^ | 0 | 282.2559 | FA18:1 | C_18_H_34_O_2_ | 3 | LC- |
| 18.73 | 4.6 | 0.63 | 0.002 | - | [M-H]^-^ | 1 | 266.2246 | FA17:2 | C_17_H_30_O_2_ | 3 | LC- |
| 23.12 | 1.8 | 2.63 | 0.002 | - | [M-H]^-^ | 0 | 296.2351 | FA18:2;O | C_18_H_32_O_3_ | 3 | LC- |
| 22.17 | 10.0 | 1.45 | 0.009 | - | [M-H]^-^ | 1 | 256.2402 | FA16:0 | C_16_H_32_O_2_ | 3 | LC- |
| 22.33 | 5.8 | 0.66 | 0.015 | - | [M-H]^-^ | 0 | 306.2559 | FA20:3 | C_20_H_34_O_2_ | 3 | LC- |
| 20.8 | 9.9 | 0.71 | 0.026 | - | [M-H]^-^ | 1 | 328.2402 | FA22:6 | C_22_H_32_O_2_ | 2 | LC- |
| 23 | 8.3 | 0.73 | 0.041 | - | [M-H]^-^ | 1 | 330.2559 | FA22:5 | C_22_H_34_O_2_ | 3 | LC- |
| 17.36 | 17.0 | 2.23 | 0.002 | 1.46 | [M+H]^+^ | 5 | 310.2508 | FA19:2;O | C_19_H_34_O_3_ | 2 | LC+ |
| 18.56 | 14.0 | 0.54 | 0.002 | 1.48 | [M+H]^+^ | 4 | 322.251 | FA20:3;O | C_20_H_34_O_3_ | 3 | LC+ |
| 20.46 | 12.6 | 0.68 | 0.002 | 1.49 | [M+H]^+^ | 5 | 336.2664 | FA21:3;O | C_21_H_36_O_3_ | 2 | LC+ |
| 20.46 | 9.2 | 0.50 | 0.009 | 1.31 | [M+H]^+^ | 5 | 262.2297 | FAL18:3 | C_18_H_30_O | 3 | LC+ |
| 21.01 | 15.7 | 4.59 | 0.002 | 1.61 | [M+H]^+^ | 5 | 324.2665 | FA20:2;O | C_20_H_36_O_3_ | 3 | LC+ |
| 24.28 | 9.9 | 3.63 | 0.002 | 1.62 | [M+H]^+^ | 4 | 340.2614 | FA20:2;O2 | C_20_H_36_O_4_ | 3 | LC+ |
| 20.706 | 2.9 | 0.66 | 0.002 | - | - | - | 284.27153 | FA18:0 | C_18_H_36_O_2_ | 1 | GC |
| 9.89 | 4.3 | 1.50 | 0.041 | - | - | - | 144.11503 | FA8:0 | C_8_H_16_O_2_ | 1 | GC |
| *Fatty aldehydes* | | | | | | | | | | | |
| 22.66 | 11.4 | 0.70 | 0.026 | 1.11 | [M+H]^+^ | 6 | 268.2766 | FAL18:0 | C_18_H_36_O | 3 | LC+ |
| *Fatty amides* | | | | | | | | | | | |
| 21.47 | 19.8 | 3.97 | 0.002 |  | [M+Cl]^-^ | 1 | 413.3141 | Stearoyl glutamic acid | C_23_H_43_NO_5_ | 3 | LC- |
| 16.45 | 2.8 | 3.24 | 0.002 | 1.64 | [M+H]^+^ | 6 | 343.2723 | Palmitoyl serine | C_19_H_37_NO_4_ | 3 | LC+ |
| 20.71 | 10.2 | 5.13 | 0.002 | 1.54 | [M+H-H_2_O]^+^ | 0 | 467.3036 | Arachidonoyl tyrosine | C_29_H_41_NO_4_ | 4 | LC+ |
| 21.21 | 0.7 | 1.61 | 0.002 | 1.59 | [M+H-H_2_O]^+^ | 5 | 429.3243 | Oleoyl phenylalanine | C_27_H_43_NO_3_ | 4 | LC+ |
| *Fatty esters* | | | | | | | | | | | |
| 21.22 | 10.2 | 4.00 | 0.002 | - | [M+Cl]^-^ | 1 | 457.3403 | CAR18:1;O2 | C_25_H_47_NO_6_ | 3 | LC- |
| 20.69 | 15.7 | 4.95 | 0.002 | - | [M+Cl]^-^ | 1 | 413.3141 | CAR16:1;O | C_23_H_43_NO_5_ | 3 | LC- |
| 1.49 | 2.3 | 0.70 | 0.002 | 1.44 | [M+H]^+^ | 3 | 203.1158 | CAR2:0 | C_9_H_17_NO_4_ | 2 | LC+ |
| 1.92 | 3.1 | 0.37 | 0.002 | 1.65 | [M+H]^+^ | 5 | 243.1471 | CAR5:1 | C_12_H_21_NO_4_ | 2 | LC+ |
| 2.05 | 0.8 | 2.31 | 0.002 | 1.65 | [M+H]^+^ | 5 | 245.1627 | CAR5:0 | C_12_H_23_NO_4_ | 2 | LC+ |
| 3.06 | 4.5 | 0.72 | 0.026 | 1.19 | [M+H]^+^ | 5 | 259.1784 | CAR6:0 | C_13_H_25_NO_4_ | 3 | LC+ |
| 16.45 | 2.8 | 3.24 | 0.002 | 1.64 | [M+H]^+^ | 6 | 343.2723 | CAR12:0 | C_19_H_37_NO_4_ | 3 | LC+ |
| 16.75 | 15.9 | 0.37 | 0.002 | 1.58 | [M+K]^+^ | 4 | 411.2985 | CAR16:2;O | C_23_H_41_NO_5_ | 3 | LC+ |
| 20.43 | 0.9 | 7.58 | 0.002 | 1.67 | [M+H]^+^ | 5 | 371.3036 | CAR14:0 | C_21_H_41_NO_4_ | 2 | LC+ |
| 21.51 | 3.7 | 17.51 | 0.002 | 1.67 | [M+H]^+^ | 5 | 415.3298 | CAR16:1 | C_23_H_45_NO_5_ | 2 | LC+ |
| 21.57 | 10.2 | 5.82 | 0.002 | 1.65 | [M+H-H_2_O]^+^ | 5 | 356.2927 | lactic stearoyl acid | C_21_H_40_O_4_ | 4 | LC+ |
| 22.76 | 0.6 | 7.25 | 0.002 | 1.65 | [M+H]^+^ | 6 | 423.3349 | CAR18:2 | C_25_H_45_NO_4_ | 2 | LC+ |
| 24.28 | 1.5 | 13.88 | 0.002 | 1.67 | [M+H]^+^ | 5 | 399.3349 | CAR16:0 | C_23_H_45_NO_4_ | 2 | LC+ |
| 25.15 | 3.1 | 15.63 | 0.002 | 1.67 | [M+H]^+^ | 5 | 425.3505 | CAR18:1 | C_25_H_47_NO_4_ | 2 | LC+ |
| ***Sphingolipids*** | | | | | | | | | | | |
| *Phosphosphingolipids* | | | | | | | | | | | |
| 18.26 | 3.5 | 2.90 | 0.002 | - | [M-H]^-^ | 0 | 379.2488 | Sphingosine-1-phosphate | C_18_H_38_NO_5_P | 3 | LC- |
| 9.19 | 12.9 | 3.90 | 0.002 | 3.25 | [M-H]^-^ | 1 | 781.5469 | CER 34:0;O2 | C_40_H_80_NO_11_P | 2 | HILIC- |
| 9.29 | 17.5 | 5.12 | 0.002 | 1.78 | [M-H]^-^ | 2 | 807.5626 | CER 36:1;O2 | C_42_H_82_NO_11P_ | 2 | HILIC- |
| 9.52 | 1.6 | 0.48 | 0.002 | 1.38 | [M-H]^-^ | 1 | 751.5 | CER 32:1;O2 | C_38_H_74_NO_11_P | 2 | HILIC- |
| *Sphingoid bases* | | | | | | | | | | | |
| 16.23 | 3.4 | 2.60 | 0.002 | 1.60 | [M+H]^+^ | 6 | 287.2824 | Sphinganine | C_17_H_37_NO_2_ | 3 | LC+ |
| 18.39 | 7.3 | 1.85 | 0.002 | 1.47 | [M+H]^+^ | 6 | 317.293 | Hydroxysphinganine | C_18_H_39_NO_3_ | 3 | LC+ |
| 19.74 | 3.5 | 1.95 | 0.002 | 1.49 | [M+H]^+^ | 6 | 299.2824 | Sphingosine | C_18_H_37_NO_2_ | 3 | LC+ |
| 22.65 | 11.2 | 0.70 | 0.015 | 1.12 | [M+H]^+^ | 6 | 285.3032 | Deoxysphinganine | C_18_H_39_NO | 3 | LC+ |
| ***Steroidal lipids*** | | | | | | | | | | | |
| 21.9 | 3.7 | 1.86 | 0.004 | 1.45 | [M+H]^+^ | 4 | 306.2195 | ST 19:1;O3 | C_19_H_30_O_3_ | 3 | LC+ |
| 1.61 | 0.8 | 8.43 | 0.002 | 1.46 | [M-H]^-^ | 2 | 452.3502 | ST 27:0;O5 | C_27_H_48_O_5_ | 3 | HILIC- |
| 1.64 | 1.6 | 35.04 | 0.002 | 2.06 | [M-H]^-^ | 2 | 420.3603 | ST 27:0;O3 | C_27_H_48_O_3_ | 3 | HILIC- |
| ***Glycerolipids*** | | | | | | | | | | | |
| *Monoacylglycerols* | | | | | | | | | | | |
| 21.9 | 1.0 | 1.86 | 0.009 | 1.34 | [M+H]^+^ | 5 | 350.2457 | MG18:4 | C_21_H_34_O_4_ | 3 | LC+ |
| 22.81 | 12.9 | 0.62 | 0.002 | 1.38 | [M+H-H_2_O]^+^ | 4 | 404.2927 | MG22:5 | C_25_H_40_O_4_ | 4 | LC+ |
| 25.3 | 3.0 | 0.85 | 0.015 | 1.04 | [M+H]^+^ | 1 | 394.2719 | MG20:4;O | C_23_H_38_O_5_ | 2 | LC+ |
| 26.51 | 8.2 | 1.45 | 0.002 | 1.43 | [M+H]^+^ | 5 | 354.277 | MG18:2 | C_21_H_38_O_4_ | 3 | LC+ |
| 26.96 | 4.2 | 3.55 | 0.002 | 1.56 | [M+H-H_2_O]^+^ | 5 | 356.2927 | MG18:1 | C_21_H_40_O_4_ | 2 | LC+ (GC) |
| 25.004 | 6.2 | 1.11 | - | 1.51 | - | - | 358.30831 | MG18:0 | C_21_H_42_O_4_ | 1 | GC |

LPC: lysophosphatidylcholine. LPE: Lysophosphatidylethanolamine. LPI: Lysophosphatidylinositols. LPS: lysophosphatidylserine. LPG: lysophosphatidylglycerol. FA: Fatty acids. CAR: Carnitines. MG: monoacylglycerols. CER: Ceramides. ST: Steroidal lipids

*The annotation of the metabolites was classified according to what was suggested by (Blaženović et al., 2018)

**Supplementary Table 2.** Compounds of non-lipid nature that presented statistically significant differences between treated and untreated parasites, identified by untargeted metabolomics using the four analytical platforms.

| **R.T.** | **% CV QC** | **F.C.** | ***P value*** | **VIP** | **Add.** | **M.E.** | **M.W.** | **ID.** | **Chem. Form.** | **ID.Level*** | **Plat.** |
| --- | --- | --- | --- | --- | --- | --- | --- | --- | --- | --- | --- |
| *Pyrimidine nucleotides* | | | | | | | | | | | |
| 1.63 | 17.4 | 0.41 | 0.002 | 1.48 | [M-H]^-^ | 0 | 607.0816 | UDP- acetylgalactosamine | C_17_H_27_N_3_O_17_P_2_ | 2 | LC- |
| 1.64 | 8.6 | 0.63 | 0.002 | 1.34 | [M-H]^-^ | 0 | 566.055 | UDP-galactose | C_15_H_24_N_2_O_17_P_2_ | 2 | LC- |
| 1.71 | 16.7 | 0.56 | 0.009 | - | [M-H]^-^ | 1 | 404.0022 | Uridine diphosphate | C_9_H_14_N_2_O_12_P_2_ | 2 | LC- |
| *Deoxyribonucleosides* | | | | | | | | | | | |
| 1.24 | 3.3 | 0.74 | 0.004 | 1.32 | [M+H]^+^ | 3 | 297.0896 | Methylthioadenosine | C_11_H_15_N_5_O_3_S | 3 | LC+ |
| *Purine nucleosides* | | | | | | | | | | | |
| 1.24 | 2.6 | 0.75 | 0.009 | 1.22 | M+H-H_2_O | 2 | 267.0968 | Adenosine | C_10_H_13_N_5_O_4_ | 3 | LC+ |
| *Carboxylic acids and derivatives* | | | | | | | | | | | |
| 1.21 | 6.6 | 0.41 | 0.002 | - | [M-H]^-^ | 1 | 721.2887 | Trypanothione | C_27_H_47_N_9_O_10_S_2_ | 3 | LC± |
| 1.87 | 6.5 | 0.50 | 0.002 | 1.49 | [M+H]^+^ | 4 | 115.0633 | Proline | C_5_H_9_NO_2_ | 2 | LC+ |
| 1.89 | 7.6 | 0.56 | 0.002 | 1.60 | [M+H]^+^ | 3 | 212.1161 | Pro Pro | C_10_H_16_N_2_O_4_ | 1 | LC+ (GC) |
| 2.19 | 2.2 | 0.50 | 0.002 | 1.59 | [M+H]^+^ | 4 | 262.1317 | Pro Phe | C_14_H_18_N_2_O_3_ | 3 | LC+ |
| 23.41 | 5.7 | 1.41 | 0.041 | 1.13 | [M+K]^+^ | 5 | 145.1103 | Aminoheptanoic acid | C_7_H_15_NO_2_ | 2 | LC+ |
| 13.91 | 3.3 | 0.66 | 0.002 | 1.41 | [M-H]^-^ | 4 | 117.079 | Aminopentanoic acid | C_5_H_11_NO_2_ | 3 | HILIC- |
| 9.173 | 6.5 | 0.44 | 0.002 | - | - | - | 102.068 | Hydroxyisovaleric acid | C_5_H_10_O_3_ | 1 | GC |
| 10.594 | 8 | 0.74 | 0.009 | 4.73 | - | - | 75.07 | Glycine | C_2_H_5_NO_2_ | 1 | GC |
| 8.169 | 8.4 | 1.07 | - | 2.98 | - | - | 90.035 | Oxalic acid | C_2_H_2_O_4_ | 1 | GC |
| 9.292 | 16.1 | 0.58 | 0.002 | 2.56 | - | - | 117.146 | Valine | C_5_H_11_NO_2_ | 1 | GC |
| 6.471 | 20 | 3.82 | 0.002 | 1.82 | - | - | 103.0630 | Ethylglycine | C_4_H_9_NO_2_ | 1 | GC |
| 10.403 | 5.4 | 0.70 | - | 1.59 | - | - | 131.0946 | Isoleucine | C_6_H_13_NO_2_ | 1 | GC |
| 12.193 | 9.7 | 1.17 | - | 1.56 | - | - | 89.0476 | Alanine | C_3_H_7_NO_2_ | 1 | GC |
| 10.093 | 12.9 | 1.66 | - | 1.45 | - | - | 131.0946 | Leucine | C_6_H_13_NO_2_ | 1 | GC |
| 13.455 | 2.5 | 1.73 | 0.041 | 1.35 | - | - | 146.0458 | Glutamate | C_5_H_9_NO_4_ | 1 | GC |
| 12.782 | 6.6 | 0.63 | 0.004 | 1.19 | - | - | 119.0218 | Aminomalonic Acid | C_3_H_5_NO_4_ | 1 | GC |
| 11.335 | 6.5 | 0.61 | 0.002 | - | - | - | 105.0425 | Serine | C_3_H_7_NO_3_ | 1 | GC |
| 2.19 | 2.6 | 0.47 | 0.002 | 1.59 | [M-H]^-^ | 4 | 359.1845 | Pro Pro Phe | C_19_H_25_N_3_O_4_ | 2 | LC± |
| *Hydroxy acids and derivatives* | | | | | | | | | | | |
| 8.442 | 4.7 | 0.23 | 0.002 | 3.45 | - | - | 104.0473 | Hydroxybutyric acid | C_4_H_8_O_3_ | 1 | GC |
| 6.949 | 7.4 | 1.09 | - | 1.21 | - | - | 89.0238 | Lactate | C_3_H_6_O_3_ | 1 | GC |
| *Keto acids and derivatives* | | | | | | | | | | | |
| 6.772 | 8.7 | 0.64 | 0.026 | - | - | - | 87.055 | Pyruvic acid | C_3_H_4_O_3_ | 1 | GC |
| *Organic phosphoric acids and derivatives* | | | | | | | | | | | |
| 14.391 | 15.6 | 0.62 | 0.009 | - | - | - | 167.9823 | Phosphoenolpyruvic acid | C_3_H_5_O_6_P | 1 | GC |
| ***Oxygenated organic compounds*** | | | | | | | | | | | |
| 19.68 | 5.0 | 1.43 | 0.002 | 2.92 | - | - | 180.0633 | Myo-Inositol | C_6_H_12_0_6_ | 1 | GC |
| 16.766 | 15.4 | 0.68 | 0.041 | - | - | - | 169.9980 | Glycerate 3 phosphate | C_3_H_7_O_7_P | 1 | GC |
| 10.937 | 5.0 | 0.71 | 0.026 | - | - | - | 106.0773 | Glyceric acid | C_3_H_6_O_4_ | 1 | GC |
| 17.805 | 13.5 | 0.74 | 0.041 | - | - | - | 180.0633 | Allose | C_6_H_12_O_6_ | 1 | GC |
| ***Others*** | | | | | | | | | | | |
| *Benzenoids* | | | | | | | | | | | |
| 1.93 | 13.1 | 0.61 | 0.002 | - | [M-H]^-^ | 5 | 136.0524 | Benzoic acid derivative | C_8_H_8_O_2_ | 2 | LC- |
| *Tetrapyrroles and derivatives* | | | | | | | | | | | |
| 20.46 | 13.1 | 0.68 | 0.002 | - | [M-H]^-^ | 10 | 562.258 | Protoporphyrin IX | C_34_H_34_N_4_O_4_ | 3 | LC- |
| *Organic Nitrogen Compounds* | | | | | | | | | | | |
| 1.48 | 2.1 | 0.55 | 0.002 | 1.45 | [M+H]^+^ | 4 | 161.1053 | Carnitine | C_7_H_15_NO_3_ | 2 | LC+ |
| *Imidazopyrimidines* | | | | | | | | | | | |
| 4.02 | 3.4 | 0.23 | 0.002 | 1.81 | [M-H]^-^ | 5 | 152.0334 | Xanthine | C_5_H_4_N_4_O_2_ | 2 | HILIC- |
| 5.37 | 2.6 | 0.35 | 0.002 | 1.66 | [M-H]^-^ | 4 | 136.0385 | HipoXanthine | C_5_H_4_N_4_O | 2 | HILIC- |

*****The annotation of the metabolites was classified according to what was suggested by (Blaženović et al., 2018)

**Reference**

Blaženović, I., Kind, T., Ji, J., and Fiehn, O. (2018). Software tools and approaches for compound identification of LC-MS/MS data in metabolomics. *Metabolites* 8. doi: 10.3390/metabo8020031.
